# Tumour necrosis factor alpha-induced neuronal loss is mediated by microglial phagocytosis

**DOI:** 10.1016/j.febslet.2014.05.046

**Published:** 2014-08-25

**Authors:** Urte Neniskyte, Anna Vilalta, Guy C. Brown

**Affiliations:** a Department of Biochemistry, University of Cambridge, UK; b Mouse Biology Unit, European Molecular Biology Laboratory, Italy

**Keywords:** cRGD, cyclic peptide arginine–glycine–aspartate–d-phenylalanine–valine, LPS, lipopolysaccharide, MFG-E8, milk fat globule EGF factor-8, MRS, MRS2578 compound, TNF-α, tumour necrosis factor-α, VNR, vitronectin receptor, Neuroinflammation, Phagoptosis, Lactadherin, Vitronectin receptor, Neurodegeneration

## Abstract

Tumour necrosis factor-α (TNF-α) is a pro-inflammatory cytokine, expressed in many brain pathologies and associated with neuronal loss. We show here that addition of TNF-α to neuronal–glial co-cultures increases microglial proliferation and phagocytosis, and results in neuronal loss that is prevented by eliminating microglia. Blocking microglial phagocytosis by inhibiting phagocytic vitronectin and P2Y_6_ receptors, or genetically removing opsonin MFG-E8, prevented TNF-α induced loss of live neurons. Thus TNF-α appears to induce neuronal loss via microglial activation and phagocytosis of neurons, causing neuronal death by phagoptosis.

## Highlights

• TNF-α induces neuronal loss in culture without neuronal necrosis or apoptosis.
• TNF-α induces microglial phagocytosis, and the neuronal loss requires microglia.
• Neuronal loss requires the microglial vitronectin receptor, P2Y_6_ receptor and MFG-E8.
• Blocking these phagocytic receptors leaves viable neurons.
• TNF-α induced phagoptosis might contribute to neuroinflammatory pathologies.

## 1 Introduction

Tumour necrosis factor-α (TNF-α) is a pro-inflammatory cytokine, central to the regulation of inflammation in body and brain. In the healthy brain, it is expressed at very low levels by a variety of brain cells including neurons [29], but microglia (brain macrophages) and astrocytes, activated by pathogens or damage via pattern recognition receptors such as Toll-like receptors (TLRs), express and release high levels of TNF-α [18], [13], [33], [21]. Thus the damaged or diseased brain contains high levels of TNF-α, including during brain trauma, ischemia, and neurodegenerative conditions [8], [22]. TNF-α activates microglia and astrocytes via TNF receptors 1 (TNFR1) and 2 (TNFR2) [11], which activate the phagocyte NADPH oxidase (PHOX) [20], caspase-8 [2] and NF-κB [5], resulting in the expression of further pro-inflammatory and phagocytic genes that increase the ability of glia to deal with pathogens and damage [31]. However, high and sustained levels of TNF-α can lead to neuronal damage [10], and there is evidence that TNF-α contributes to a variety of brain pathologies, such as ischemic stroke, Alzheimer’s disease, Parkinson’s disease, and multiple sclerosis [8], [22]. The mechanisms by which TNF-α is damaging to neurons is unclear, but the inflammatory activation of glia may contribute.

In culture, we have found that activation of microglial TLRs results in high levels of TNF-α production [13], [23], [15] and TNF-α in turn stimulates microglial proliferation [20]. We subsequently found that activation of microglia via TLR2 or TLR4 caused delayed neuronal loss via activating microglial phagocytosis of stressed-but-viable neurons [23]. Cell death caused by the cell being phagocytosed is called ‘phagoptosis’ with the defining characteristic that inhibition of phagocytosis or phagocytic signalling prevents death of the cell [6]. TLR activation of microglia causes: (i) release of oxidants from microglia that induce neurons to reversibly expose the ‘eat-me’ signal phosphatidylserine, (ii) release of the opsonin milk fat globule EGF factor-8 (MFG-E8) from microglia and astrocytes that binds exposed phosphatidylserine on neurons and activates their phagocytosis via the microglial vitronectin receptor (VNR), and (iii) activation of the phagocytic capacity of microglia via upregulation of Mer tyrosine kinase and other phagocytic proteins [23], [24], [7].

As activation of microglia can cause both TNF-α production and neuronal phagoptosis, we tested here whether soluble, extracellular TNF-α was able and sufficient to induce microglia-dependent neuronal loss by phagoptosis.

## 2 Materials and methods

### 2.1 Reagents

Rat recombinant TNF-α (Sigma), lipopolysaccharide from *Salmonella typhimurium* (Sigma), Alexa Fluor 488 conjugate of isolectin B_4_ from *Griffonia simplicifolia* (Invitrogen), carboxylate-modified fluorescent microspheres (Invitrogen), cyclo(RGDfV) peptide (Bachem), recombinant MFG-E8 (R&D Systems), human soluble TNF receptor inhibitor/Fc chimera (GenScrip Corporation), and MRS2578 compound (Tocris). Cell culture reagents were from PAA. Other reagents were from Sigma.

### 2.2 Primary cell culture and treatment

All experiments were performed in accordance with the UK Animals (Scientific Procedures) Act (1986) and approved by the Cambridge University local ethical committee. Primary mixed neuronal/glial cultures from postnatal days 5–7 Wistar rat or *Mfge8^−/−^* mice [30] cerebella were prepared as described previously [16]. Cells were plated at a density of 5 × 10^5^ cells/well on poly-l-lysine coated 24-well plates. Glial cultures and pure microglial cultures were prepared as described previously [3]. Microglia cells were depleted from the cultures with l-leucine methyl ester as described previously [23]. Cells were stimulated at 7–9 days in vitro with TNF-α (50 ng/ml) or LPS (100 ng/ml), and cyclo(RGDfV) peptide (50 μM), recombinant MFG-E8 (0.4 μg/ml) or soluble TNF receptor inhibitor (100 ng/ml) were added together with TNF-α, whereas MRS2578 compound (1 μM) was added every day. Cell densities after treatment were evaluated as described previously [27]. Neurons with regular soma shape and normal nuclear Hoechst 33342 staining were counted as alive, neurons with condensed chromatin were considered as apoptotic, whereas neurons staining with propidium iodide were defined as necrotic.

### 2.3 Microglial phagocytosis of beads

Phagocytic capacity of microglial cells was evaluated as described previously [27]. In short, pure microglial culture was treated with 50 ng/ml TNF-α for 24 h before 3 μl of 1:10 dilution of 1 μm fluorescently labelled carboxylate-modified microspheres were added, and cells were incubated for 2 h at 37 °C, 5% CO_2_. The medium was removed, and the culture was washed several times to remove excess beads. Microglia cells were then labelled with Alexa Fluor 488-tagged isolectin B_4_ (2 μg/ml) and bead number per cell was evaluated in >50 cells per condition.

### 2.4 Microglial phagocytosis of neurons

Glial cultures were treated ± 50 ng/ml of TNF-α for 24 h. Microglia from untreated and treated flasks were detached from other glia by shacking the flask. 10^5^ microglia (untreated and treated) were added to each well (in a 24 well plate) of a mixed neuronal/glial culture, which had previously been stained for 15 min with TAMRA (red fluorescence) and washed. Phagocytosis was assayed in a medium half from a glial culture and half from a mixed neuronal/glial culture. Phagocytosis of neurons by microglia was evaluated by microscopy at 6 h after adding microglia as the number of microglia per field containing red fluorescent debris. Cells were also stained with Hoechst 33342 (for nuclei) and green fluorescent isolectin B_4_ (for microglia).

### 2.5 Statistical analysis

For all experiments, each condition/treatment was repeated at least in duplicate, and each experiment was replicated in at least three independent cultures – except the experiment on microglial phagocytosis of neurons, which was repeated in quadruplicate but on one culture. Statistical analysis was performed using IBM SPSS Statistics v20 software. Normality of data was verified by Shapiro–Wilk test. Means were compared by one-way ANOVA, and the significance of the difference between each treatment mean and the control or TNF-α treatment mean was quantified by *post-hoc* Bonferroni tests. All such significant changes are reported in the Figures – those not reported as significant are not significant. *P* values < 0.05 were considered as significant. Numbers of alive, apoptotic and necrotic neurons were compared separately. All data presented are expressed as mean ± standard error of the mean (S.E.M.).

## 3 Results and discussion

**Fig. 1:**
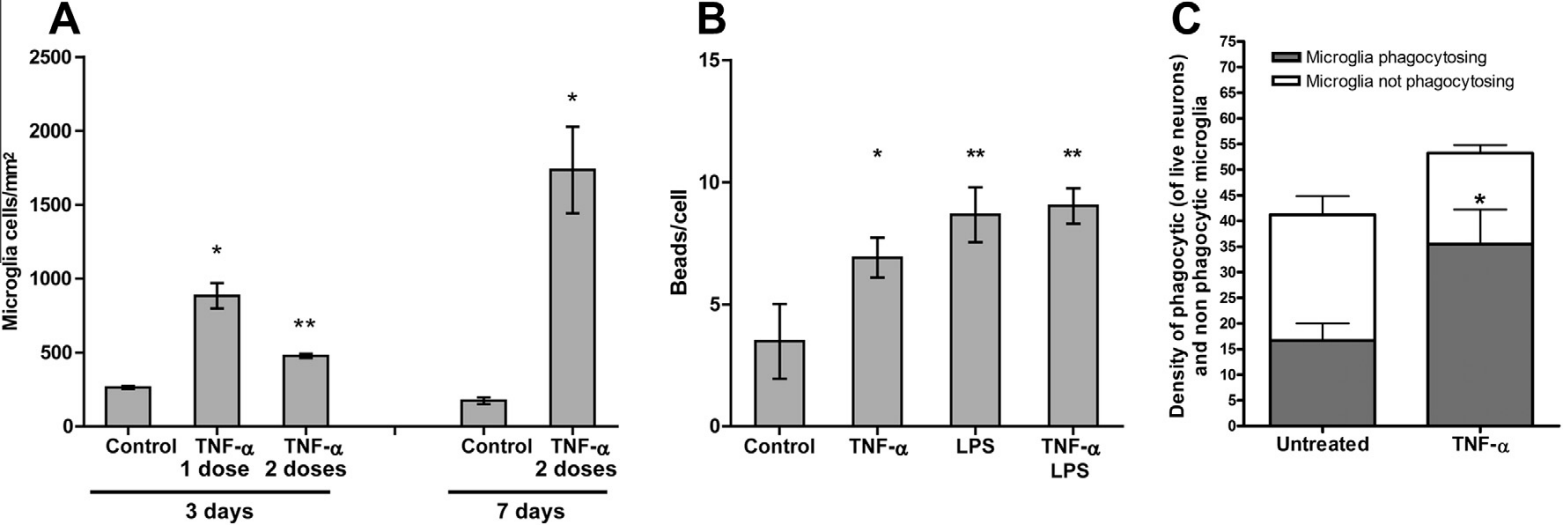
TNF-α stimulates microglial proliferation and phagocytosis. (A) Treatment with TNF-α, added either as a single dose (50 ng/ml) or two doses (second dose of 50 ng/ml added 24 h after the first) significantly increase microglial density in mixed neuronal/glial cultures, 3 or 7 days after the first addition of TNF-α. (B) Addition of TNF-α (50 ng/ml for 24 h) increases the phagocytic uptake of fluorescent 1 μm carboxylate-modified microspheres in pure microglial culture, but does not have a synergistic or antagonistic effect with lipopolysaccharide (*LPS*, 100 ng/ml). (C) Pre-treatment of microglia with TNF-α (50 ng/ml for 24 h) increased the proportion of microglia that phagocytosed neurons when the microglia were added to a healthy neuronal–glial culture for 6 h. The numbers of microglia per field that either contained or did not contain neuronal debris are quantified. Data are presented as means ± S.E.M. of ⩾3 cultures (A and B) or of 4 replicates on the same culture (C). ^∗/^^∗∗^*P* < 0.05/0.01 *versus* untreated control.

**Fig. 2:**
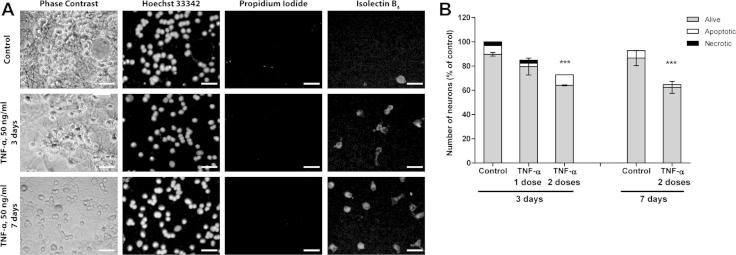
TNF-α induces neuronal loss in mixed cultures. (A) Representative images of culture treated for 3 or 7 days with two doses of TNF-α (50 ng/ml, second dose added 24 h after the first one). Cell nuclei are labelled with Hoechst 33342, necrotic cells are labelled with propidium iodide, microglia cells are labelled with isolectin B_4_. *Scale bars*, 25 μm. (B) One dose of TNF-α (50 ng/ml) is not sufficient to induce neuronal loss within 3 days of treatment. Two doses of TNF-α (second dose of 50 ng/ml added 24 h after the first one) induce significant neuronal loss within first 3 days of treatment. No more neurons are lost for up to 7 days. Data are presented as means for ⩾3 independent experiments; ± S.E.M. of live neurons; ^∗∗∗^*P *< 0.001 *versus* control.

**Fig. 3:**
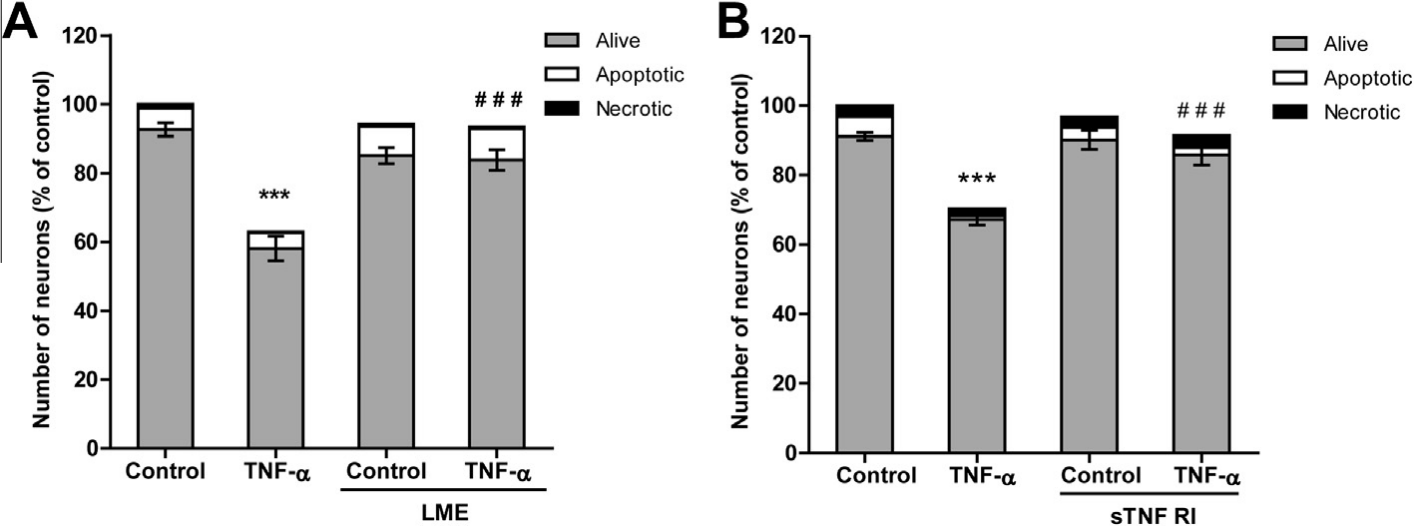
TNF-α induced neuronal loss requires microglia, and is prevented by a TNFR inhibitor. (A) Neuronal loss induced by TNFα is prevented by selective depletion of microglial cells with l-leucine-methyl ester (*LME*). (B) Soluble TNF receptor inhibitor (*sTNF RI*, 100 ng/ml) prevents neuronal loss induced by TNF-α. (B) Data are presented as means for ⩾3 independent experiments, ± S.E.M. of live neurons; ^∗∗∗^*P* < 0.001 *versus* control, ^###^*P* < 0.001 *versus* TNF-α.

TNF-α (50 ng/ml, equivalent to 3 nM of monomer) caused microglial proliferation (Figs. 1A and 2A), stimulated microglial phagocytosis of beads (Fig. 1B), and increased the phagocytosis of neurons by added microglia (Fig. 1C). However, in mixed neuronal–glial cultures, a single dose of TNF-α was not sufficient to cause significant neuronal loss (Fig. 2B). As extracellular TNF-α is rapidly removed/degraded [15], we tested whether significant neuronal loss could be induced by a second bolus of 50 ng/ml TNF-α added 24 h after the first dose, and cultures were incubated for a further 2 or 6 days (in total 3 or 7 days treatment, respectively). Two doses of TNF-α were sufficient to induce significant neuronal loss after 3 days of treatment (Fig. 2A and B). There was no further loss of neurons for up to 7 days (Fig. 2B), even though prolonged treatment with TNF-α increased microglial densities by up to ten times (Fig. 1A). This is in accordance with previously published data demonstrating that phagoptosis induced with different stimuli is maximal 2–3 days after culture stimulation [23], [27]. Higher concentration (100 ng/ml) of TNF-α did not further increase microglial numbers or neuronal loss (data not shown). Adding soluble TNF receptor inhibitor to chelate extracellular TNF-α prevented the neuronal loss induced by TNF-α (Fig. 3B), indicating that loss was indeed due to TNF-α rather than some contaminant such as endotoxin.

Treatment with TNF-α did not increase the number of apoptotic or necrotic neurons in the neuronal glial cultures (Fig. 2A and B), suggesting that TNF-α was not directly toxic to the neurons. Therefore we tested whether eliminating microglia from the cultures by treating them with l-leucine methyl ester [23] would prevent TNF-α-induced neuronal loss. There was no neuronal death or loss in microglia-deficient cultures stimulated with TNF-α (Fig. 3A), indicating that TNF-α was not directly toxic to neurons, and the neuronal loss required the presence of microglia.

**Fig. 4:**
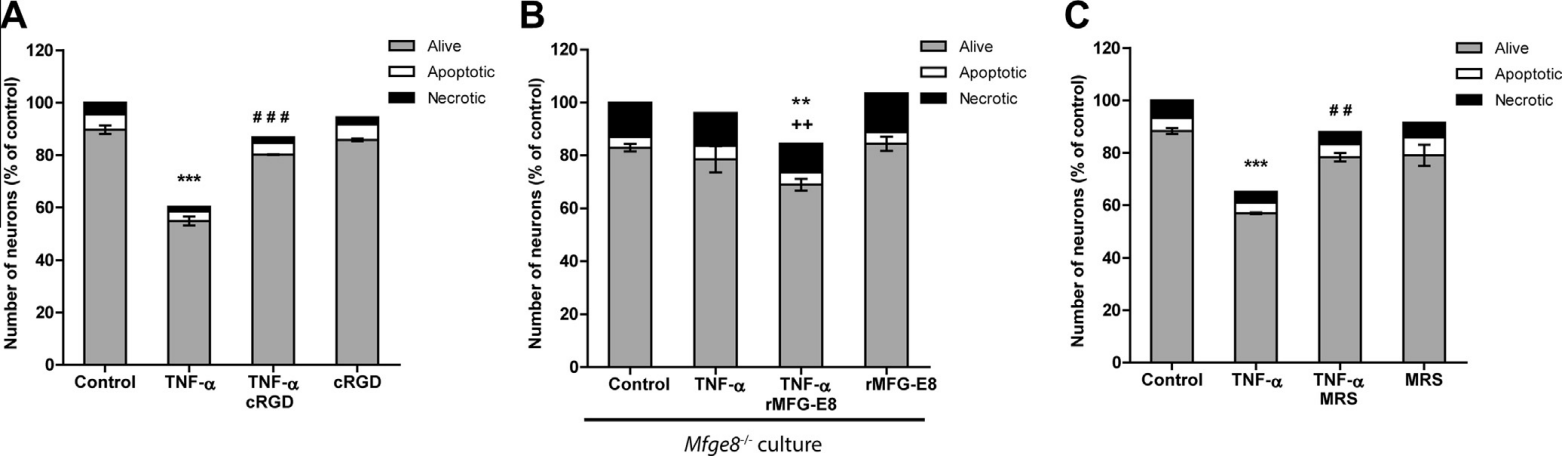
Inhibition of phagocytic signalling prevents neuronal loss and death induced by TNF-α. (A) Loss of neurons induced by TNF-α (two doses of 50 ng/ml for 3 days) is prevented by blocking the phagocytosic receptor VNR with cyclo(RGDfV) peptide (*cRGD*, 50 μM). (B) TNF-α does not induce neuronal loss in neuronal/glial cultures from *Mfge8*^−/−^ mouse cerebella, but the loss is reconstituted by the addition of recombinant MFG-E8 protein (*rMFG-E8*, 0.4 μg/ml, added together with the first dose of TNF-α). (C) Neuronal loss in mixed neuronal/glial cultures treated with tumour necrosis factor-α (*TNF-α*, 50 ng/ml) for 3 days is prevented by the selective P2Y_6_ inhibitor MRS2578 (*MRS*, 1 μM, added every day). Data are presented as means for ⩾3 independent experiments, ± S.E.M. of live neurons, ^∗∗/^^∗∗∗^*P* < 0.01/0.001 *versus* control, ^##/^^###^*P* < 0.01/0.001 *versus* TNF-α, ^++^*P* < 0.01 *versus* rMFG-E8.

Microglia-induced loss of neurons may be mediated via variety of mechanisms: microglial release of glutamate to induce excitotoxicity [14], microglial neurotoxic mediators (such as reactive oxygen species) [19] that could cause neuronal apoptosis or detachment from the culture, or neuronal death executed by microglial phagocytosis. We have previously shown that microglial phagoptosis of stressed-but-viable neurons depends on neuronal exposure of phosphatidylserine [23], [27]. Exposed phosphatidylserine is bound by the soluble opsonin milk fat globule EGF factor-8 (MFG-E8), which is in turn recognized by the microglial vitronectin receptor (VNR, an α_v_β_3_ or α_v_β_5_ integrin) [28], [12], [26]. To investigate whether microglia might contribute to TNF-α-induced neuronal loss by phagocytosing viable neurons, we targeted the phagocytic MFG-E8/VNR pathway. Inhibition of phagocytosis with specific VNR inhibitor cyclo(RGDfV) peptide prevented the loss of neurons induced by TNF-α (Fig. 4A), indicating that neuronal loss induced by TNF-α was dependent on VNR.

In order to test whether the VNR-specific opsonin MFG-E8 was required for TNF-α-induced neuronal loss, we isolated neuronal–glial cultures from the cerebella of *Mfge8*^−/−^ mice [30]. The amount of necrotic neurons in these untreated cultures was higher (Fig. 4B). Addition of TNF-α induced no neuronal loss in these cultures lacking MFG-E8 (Fig. 4B). However, TNF-α-induced neuronal loss in *Mfge8*^−/−^ culture was reconstituted by adding recombinant MFG-E8 protein together with TNF-α, whereas adding MFG-E8 alone had no effect (Fig. 4B). Thus MFG-E8 is required for TNF-α-induced neuronal loss.

The microglial P2Y_6_ receptor is required for microglial phagocytosis of neurons, as UDP released from damaged neurons induces formation of the phagocytic cup via activating microglial P2Y_6_ [17]. And we have recently shown that the P2Y_6_ receptors is required for the microglial phagocytosis of neurons induced by LPS or amyloid β [25]. So we tested whether inhibition of P2Y_6_ with 1 μM MRS2578 prevented TNF-α-induced neuronal loss, and found that it did (Fig. 4C), indicating that activation of the P2Y_6_ phagocytic receptor is required for this neuronal loss.

Importantly, inhibition of P2Y_6_, inhibition of VNR or lack of MFG-E8 prevented neuronal loss without statistically significant increases of the number of apoptotic or necrotic neurons in the cultures treated with TNF-α (Fig. 4). If microglia had been phagocytosing dead or dying neurons then inhibition of their phagocytosis would have left necrotic or apoptotic neurons. Whereas we found that inhibition of phagocytosis prevented neuronal loss and left viable neurons, indicating that the neurons must have been viable at the time of their phagocytosis, i.e. the neurons died by phagoptosis. These data demonstrated that TNF-α induces neuronal loss that is mediated by microglial phagocytosis of otherwise viable neurons recognized via the MFG-E8/VNR pathway.

It has been previously established that TNF-α activates microglia and triggers NADPH oxidase activation [9], [13]. Even though TNF-α itself is not directly neurotoxic [1], it may contribute to neuronal damage due to microglial activation. Here we demonstrated that TNF-α induced strong microglial proliferation (Fig. 1A), which is often used as an indicator of microglial activation. In addition, TNF-α increased the microglial phagocytic capacity (Fig. 1B), thus promoting microglial uptake of neurons. Under inflammatory conditions TNF-α may be released by either activated microglial cells or astrocytes. While it has been previously shown that microglia cells produce TNF-α when activated with such triggers as LPS [23] or amyloid β [13], whether TNF-α levels released by microglia are sufficient to induce phagoptosis has not yet been demonstrated. TNF-α concentrations that were used in this study to induce phagoptosis were significantly higher than those found in cerebrospinal fluid of patients with neurodegenerative diseases (0.8 ng/ml in Alzheimer’s disease; [32]) or animal models of brain inflammation (0.4 ng ml in rat experimental meningitis [4]). However, it is difficult to extrapolate from these studies to what might be focal/local extracellular concentration of TNF-α in diseased brain. The relevance of TNF-α for phagoptosis in vivo is yet to be determined.

Treatment with TNF-α alone was sufficient to induce microglia-dependent neuronal loss (Fig. 2A and B). Furthermore, this loss of neurons was prevented by the VNR inhibitor cyclo(RGDfV) peptide and in *Mfge8*^−/−^ cultures, indicating that neuronal loss was mediated by the MFG-E8/VNR phagocytic pathway of microglial cells (Fig. 4A and B). Inhibition of P2Y_6_ also prevented neuronal loss indicating that this neuronal loss also required the UDP/P2Y_6_ phagocytic pathway (Fig. 4C). While pharmacological treatments used in this study may potentially have had side effects unrelated to phagocytosis (such as changes in microglial secretome), it is unlikely that such effects could have blocked neuronal death or promoted neuronal survival.

Altogether, the data presented here revealed that TNF-α induces neuronal death by microglial phagocytosis. Consequently the delayed neuronal loss that occurs in many pathologies accompanied by inflammation may be due to TNF-α-induced phagoptosis, and might be prevented by blocking TNF-α production or function, its receptor, microglial activation, MFG-E8, the vitronectin receptor or the P2Y_6_ receptor.
